# Selective ligninolysis of wheat straw and wood chips by the white-rot fungus *Lentinula edodes* and its influence on *in vitro* rumen degradability

**DOI:** 10.1186/s40104-016-0110-z

**Published:** 2016-09-22

**Authors:** Sandra J. A. van Kuijk, José C. del Río, Jorge Rencoret, Ana Gutiérrez, Anton S. M. Sonnenberg, Johan J. P. Baars, Wouter H. Hendriks, John W. Cone

**Affiliations:** 1 Animal Nutrition Group, Wageningen University, De Elst 1, 6708 WD Wageningen, The Netherlands; 2 Instituto de Recursos Naturales y Agrobiologica de Sevilla (IRNAS-CSIC), Avenida Reina Mercedes, 10, 42012 Seville, Spain; 3 Plant Breeding, Wageningen University, Droevendaalsesteeg 1, 6708 PB Wageningen, The Netherlands

**Keywords:** Fungal treatment, *In vitro* rumen degradability, Lignocellulosic biomass, Py-GC/MS

## Abstract

**Background:**

The present work investigated the influence of lignin content and composition in the fungal treatment of lignocellulosic biomass in order to improve rumen degradability. Wheat straw and wood chips, differing in lignin composition, were treated with *Lentinula edodes* for 0, 2, 4, 8 and 12 wk and the changes occurring during fungal degradation were analyzed using pyrolysis-gas chromatography-mass spectrometry and detergent fiber analysis.

**Results:**

*L. edodes* preferentially degraded lignin, with only limited cellulose degradation, in wheat straw and wood chips, leaving a substrate enriched in cellulose. Syringyl (S)-lignin units were preferentially degraded than guaiacyl (G)-lignin units, resulting in a decreased S/G ratio. A decreasing S/G ratio (wheat straw: *r* = −0.72, wood chips: *r* = −0.75) and selective lignin degradation (wheat straw: *r* = −0.69, wood chips: *r* = −0.88) were correlated with *in vitro* gas production (IVGP), a good indicator for rumen degradability.

**Conclusions:**

*L. edodes* treatment increased the IVGP of wheat straw and wood chips. Effects on IVGP were similar for wheat straw and wood chips indicating that lignin content and 3D-structure of cell walls influence *in vitro* rumen degradability more than lignin composition.

## Background

Carbohydrates in plant cell walls can be an important source of nutrients for ruminants. However, these carbohydrates are bound to lignin, which can be degraded only under aerobic conditions by fungi and some bacteria [1], and as such cannot be broken down in the low oxygen environment of the rumen. Currently, several chemical and physical pre-treatments are used to make the carbohydrates in lignocellulosic substrates more available for degradation in the rumen [2, 3]. Biological treatments using fungi that selectively degrade lignin, without or with limited cellulose degradation, may be more cost effective and less harmful for animals and the environment compared to current pre-treatment methods. Studies reported in the scientific literature describe treatments by different fungi to pre-treat various substrates suitable as ruminant feed ingredients [3]. Among them, the white rot fungus *Lentinula edodes* was found to be highly promising due to its selective lignin degradation pattern [4–10]. In addition, this fungus has a ‘generally regarded as safe’ (GRAS) status and is, therefore, a potentially suitable fungus for fungal pre-treatment of feed ingredients [4]. However, due to the GRAS status of edible mushrooms of this fungal genus, hitherto the majority of research has focused on mushroom production [6–8].

Scientific studies describing *L. edodes* treatment of lignocellulosic biomass to increase rumen degradability often report changes in lignin content as measured by the Van Soest acid detergent lignin (ADL) methodology [3–5, 11]. However, ADL does not represent the total lignin content, since acid soluble lignin is not included in the ADL fraction leading to an underestimation [12, 13]. Lignin is a complex aromatic polymer produced by the oxidative coupling of three main monolignols, sinapyl, coniferyl and *p*-coumaryl alcohols, differing in their degree of methoxylation [14]. When incorporated into the lignin polymer, these monolignols give rise to the syringyl (S), guaiacyl (G) and *p*-hydroxyphenyl (H) units, respectively, generating a variety of structures and linkages within the polymer, including β–*O*–4′ alkyl-aryl ethers, phenylcoumarans and resinols, amongst others. Lignin composition is different for each plant species, for example, lignin in grasses and herbaceous plants consists of S-, G-, and H-units, whereas softwoods present mainly G-lignin units and hardwoods present S- and G-units in different proportions [15]. Lignin composition can be rapidly assessed using pyrolysis coupled to gas chromatography-mass spectrometry (Py-GC/MS) [16]. During pyrolysis the plant material is heated at high temperatures (usually around 500–700 °C) in an oxygen-free environment to break down the macromolecular components of plant cell walls to smaller compounds, which are subsequently analyzed in a GC/MS system. As such, Py-GC/MS is a useful tool to monitor the extent of fungal degradation of lignocellulosic constituents, which cannot be straight-forwardly detected with the standard gravimetric methods, such as the detergent fiber method [16–18].

In this study it is hypothesized that in addition to the total lignin content, the lignin composition (in terms of relative abundances of the S-, G-, and H-lignin units) and the 3D-structure formed by lignin and carbohydrates in plant cell walls also determine the efficiency of the fungal treatment to improve rumen degradability. In this paper, two substrates (wheat straw and wood chips) differing in lignin content and composition were treated with *L. edodes* and analyzed for cell wall components using the detergent fiber method, and lignin composition using Py-GC/MS. Rumen degradability of treated substrates was measured by the *in vitro* gas production technique.

## Methods

### Fungal strains and spawn preparation

*Lentinula edodes* (strain MES 11910) was used in this study. The strain MES11910 originates from the CCBAS culture collection of basidiomycetes (Institute of microbiology, academy of sciences of the Czech Republic (http://www.biomed.cas.cz/ccbas/fungi.htm). The strain has been isolated in 1961 in Japan and found on Passania wood. The species identity was confirmed with ITS sequencing. *L. edodes* was cultured on malt extract agar plates at 24 °C until most of the plate surface was covered with mycelium. Pieces of colonized agar culture (~1 cm^2^) were added to sterilized sorghum grains (*Sorghum bicolor* var. *bicolor*, type red dari) and incubated at 24 °C until all grains were colonized by mycelium. This spawn was kept at 4 °C until the substrates were ready to use (~1 wk).

### Substrate preparation

The substrates used were wheat straw and municipal trimmings consisting of a mixture of chips from different wood species. Both substrates were chopped into pieces of approximately 3 cm length and submerged in tap water (pH was not adjusted) for 3 d at room temperature to allow moisture to fully penetrate. After removal of excess water, substrates were autoclaved twice with the first sterilization performed in autoclavable bags at 121 °C for 1 h. After cooling, the material was weighed into 1.2 L polypropylene containers fitted with a cover containing a filter allowing gas exchange, but preventing contamination (model TP1200 + TPD1200 XXL Combiness, Nazareth, Belgium). Each container was filled with approximately 80–90 g dry matter of wheat straw or wood chips before being autoclaved a second time at 121 °C for 1 h to kill the remaining germinated spores in the substrates. After cooling, the sterile substrates were kept in the container at room temperature until further use. Three of these autoclaved containers were used as an uninoculated control (0 wk treatment).

### Substrate inoculation

To each remaining container, approximately 8–10 g of spawn (sorghum grains colonized with a pure culture of *L. edodes*) was added and mixed to distribute the spawn equally over the substrate. Both handlings were performed aseptically. Each container was then incubated for 2, 4, 8 or 12 wk at 24 °C and 70 % relative humidity in a climate controlled chamber. All conditions were tested in triplicate.

After incubation, the substrate was air-dried at 70 °C until constant weight. The dried wheat straw was ground with a Peppink 100 AN cross beater mill (Peppink, Deventer, The Netherlands) over a 1 mm sieve. The dried wood chips were first coarsely ground over a 1 mm sieve using a Retch SM2000 cutting mill (Retch, Haan, Germany) before being ground over a 1 mm sieve using a Retch ZM 100 centrifugal mill (Retch, Haan, Germany). Samples were stored at 4 °C until chemical analyses.

### Fiber analysis

Samples were analyzed according to the Van Soest method [11]. The hemicellulose content was calculated as the difference between the neutral detergent fiber (NDF) and the acid detergent fiber (ADF). The lignin content was determined as the ‘acid detergent lignin’ (ADL) content, that was defined as the part of the cell wall that is not soluble in acid detergent reagent and 72 % sulphuric acid. The cellulose content was calculated as the difference between ADF and ADL. For dry matter (DM) determination, air-dried material was dried at 103 °C for 4 h. Ash content was determined by combustion for 3 h at 550 °C in a muffle furnace. The data for three replicate samples were averaged and expressed as g/kg DM.

### Pyrolysis-GC/MS

Pyrolysis-GC/MS (approximately 1 mg) was performed with a 3030 μ-furnace pyrolyzer (Frontier Laboratories Ltd.) connected to an Agilent 7820A GC using a DB-1701 fused-silica capillary column (60 m × 0.25 mm, 0.25 μm film thickness) and an Agilent 5975 mass selective detector (EI at 70 eV). The pyrolysis was performed at 500 °C. The oven temperature of the gas chromatograph was programmed from 100 °C (4 min) to 280 °C at a rate of 3 °C/min and held at the maximum temperature for 2 min. The transfer line was set to 290 °C. Helium was the carrier gas with a constant flow of 1 mL/min. The compounds were identified by comparing their mass spectra with those of the Wiley (John Wiley and Sons, Hoboken, NJ, USA) and NIST/EPA/NIH 2011 (National Institute of Standards and Technology, Gaithersburg, MD, USA) mass spectral libraries and those reported in literature [19, 20]. Peak areas corrected for molecular weight were calculated for the carbohydrate and lignin-degradation products, the summed areas were normalized, and the data for three replicate samples were averaged and expressed as percentages.

### *In vitro* gas production technique

The *in vitro* gas production (IVGP) technique was performed according to the procedure previously described [21]. In short, rumen fluid of 2 fistulated non-lactating cows fed a grass silage based diet was mixed with a buffer solution under anaerobic conditions. Air dried samples (500 mg) were incubated in 60 mL buffered rumen fluid (final dilution 3 times) for 72 h at 39 °C. The gas production was automatically recorded as previously described [21], and the data for three replicate samples were averaged and expressed as mL gas/g organic matter (OM).

### Statistical analysis

A generalized linear model (GLM) analysis in SAS 9.3 was used to compare fiber composition and IVGP of the fungal treatment at each incubation time to the autoclaved, uninoculated control of each substrate. The following model was used:

$$ {\mathrm{Y}}_{ij}=\upmu +{\upalpha}_i+{\upomega}_{ij} $$

in which Y_*ij*_ is the observation *j* in treatment *i*; μ is the overall mean; α_*i*_ is the fixed effect of treatment *i*; ω_*ij*_ is the random error. Post-hoc multiple comparison with Tukey’s significant test at a level of α = 0.05 was performed to determine the significance between the treatments.

Regression analysis between IVGP and fiber composition of fungal treated substrates was analyzed in SAS 9.3.

The correlation between IVGP and Py-GC/MS data was analyzed in SAS 9.3. The correlations are provided as the Pearson correlation coefficient (r).

## Results

### Composition of wheat straw and wood chips during fungal treatment

The wood chips used in the current study originated from municipal trimmings consisting of a mixture of different wood species, which all contain a different lignin composition. The composition presented in this study is therefore representative for this mixture, since both technical (each sample was measured in duplicate) and biological (each treatment was measured in triplicate) replicates are presented here.

The ADL, hemicellulose and cellulose content of wheat straw and wood chips before and after *L. edodes* treatment for 2, 4, 8 and 12 wk is shown in Table 1. Untreated wheat straw had a lower ADL, higher hemicelluloses and similar cellulose content compared to untreated wood chips. Upon *L. edodes* treatment, the content of ADL and hemicelluloses of wheat straw decreased (*P* < 0.05)*,* while the cellulose content increased (*P* < 0.05) compared to the untreated control. Expressed in absolute amounts, ADL decreased (*P* < 0.05) up to 87 %, hemicelluloses (*P* < 0.05) up to 77 % and cellulose was degraded up to 20 %, but this was not significant (*P* = 0.07) (Table 1). The amounts of dry matter significantly decreased by 30 % after 12 wk. Fungal treatment of wood chips also resulted in a decrease (*P* < 0.05) in the ADL content during the 12 wk of incubation. No significant decrease in hemicelluloses and cellulose content was observed after 12 wk incubation compared to the autoclaved control. The same applied to the absolute amounts of each compound, only ADL decreased significantly (*P* < 0.05) over time during *L. edodes* treatment of wood chips compared to the uninoculated control.

**Table 1:** Chemical composition of autoclaved wheat straw and wood chips before and after treatment with *L. edodes* for 2, 4, 8 and 12 wk

Substrate	Treatment time, wk	Contents, g/kg DM			Amounts, g			DM loss, %	IVGP, mL/g OM
		ADL	HC	Cell	ADL	HC	Cell		
Wheat straw	0	81.1^a^	260.1^a^	479.6^b,c^	7.5^a^	24.2^a^	44.6	0^c^	252.8^a,b^
	2	74.6^b^	202.7^b^	461.2^c^	6.4^b^	17.4^b^	39.5	8^c^	247.8^b^
	4	57.0^c^	148.1^c^	496.3^b^	4.7^c^	12.2^c^	41.0	11^b,c^	277.0^a,b^
	8	33.2^d^	94.6^d^	537.3^a^	2.4^d^	6.9^d^	39.1	22^b^	287.3^a,b^
	12	15.5^e^	87.2^d^	544.2^a^	1.0^e^	5.7^d^	35.6	30^a^	311.2^a^
	RMSE	2.22	11.48	11.80	0.29	1.22	3.21	0.05	22.40
	*P*-value	<0.01	<0.01	<0.01	<0.01	<0.01	0.07	<0.01	0.03
Wood chips	0	198.2^1^	140.8^a^	445.5^c^	15.4^a^	11.0^a^	35.3	0	54.0^c^
	2	187.3^a,b^	96.3^a,b^	486.2^b^	15.1^a^	7.8^a,b^	39.2	0	55.4^c^
	4	163.8^b^	103.6^a,b^	497.4^a,b^	10.1^b^	6.8^a,b^	31.1	20	120.7^b^
	8	126.6^c^	79.9^b^	520.6^a^	8.8^b^	5.6^b^	36.1	11	169.6^a^
	12	107.0^c^	105.6^a,b^	436.5^c^	6.9^b^	6.8^a,b^	28.5	17	177.4^a^
	RMSE	9.49	16.97	11.59	1.44	1.81	4.53	1.2	16.12
	*P*-value	<0.01	0.02	<0.01	<0.01	0.04	0.10	0.16	<0.01

Values with different superscripts within column are significantly (*P* < 0.05) different

*ADL* acid detergent lignin, *HC* hemicellulose, *Cell* cellulose, *DM loss* dry matter loss, *IVGP in vitro* gas production, *RMSE* root-mean-square error

### *In vitro* gas production (IVGP) of fungal treated samples

IVGP of wheat straw increased (*P* = 0.058) from 252.8 mL/g OM in the untreated control sample (0 wk treatment) to 311.2 mL/g OM after 12 wk of *L. edodes* treatment, a 23 % increase (Table 1). For fungal treated wood chips, the IVGP of the uninoculated control (0 wk treatment) was 54.0 mL/g OM and a significant increase (*P* < 0.05) was already seen after 4 wk treatment, and continued to increase during the 12 wk treatment up to a value of 177.4 mL/g OM, representing a nearly 230 % increase compared to the untreated sample (Table 1).

Regression analysis between IVGP (mL/g OM) and cell wall composition (g/kg) yielded the following equation:

$$ IVGP = 0.26\times hemicelluloses + 0.33\times cellulose-1.34\times ADL+135.86 $$

This equation indicates that changes in ADL have more effect on IVGP than hemicelluloses and cellulose. The large influence of ADL on IVGP is the reason to study lignin in more detail.

### Py-GC/MS analyses of uninoculated and fungal treated substrates

**Fig. 1:**
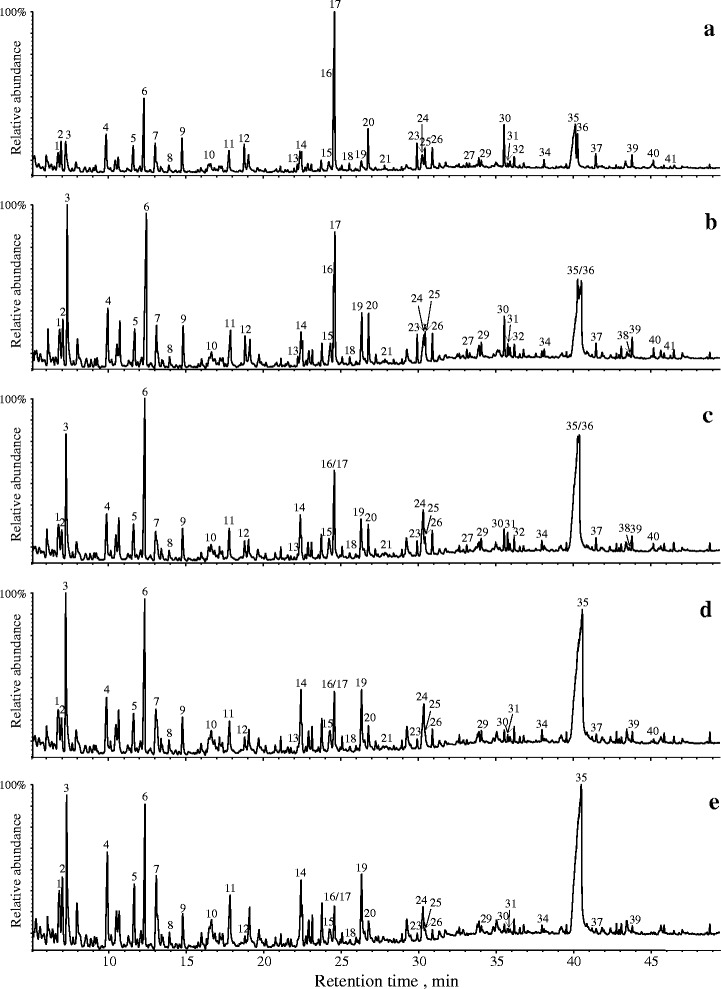
Py-GC/MS chromatograms of wheat straw degraded with the fungus *Lentinula edodes*. **a** untreated wheat straw control (0-wk incubation); **b** wheat straw degraded for 2 wk; **c** 4 wk; **d** 8 wk; **e** 12 wk. The identities and relative abundances of the compounds represented by the numbered peaks are listed in Table 2

**Fig. 2:**
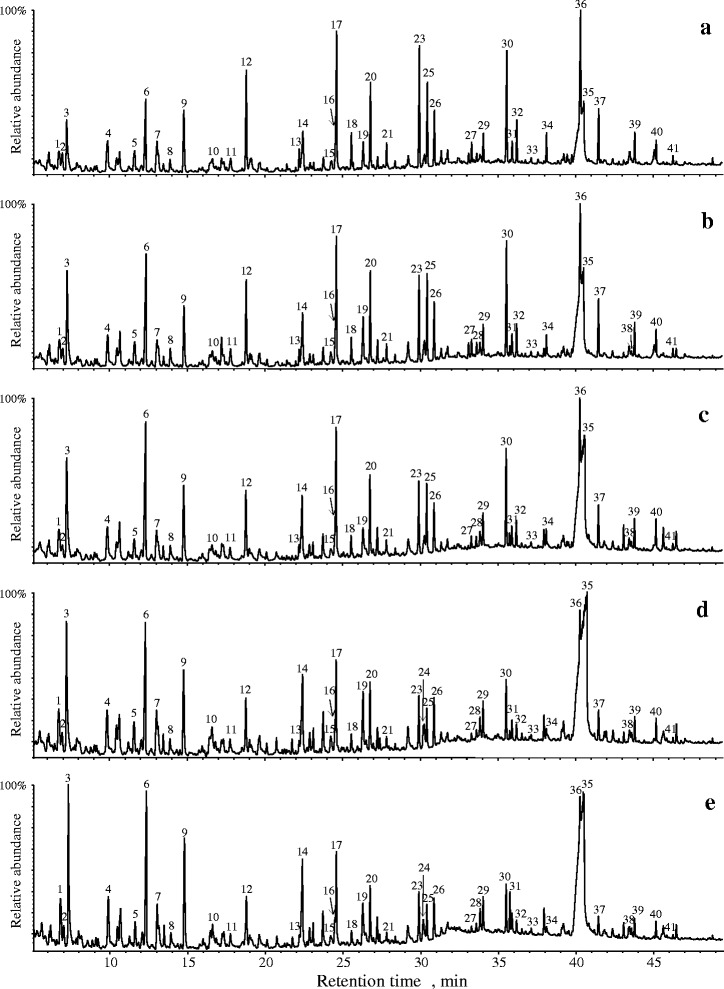
Py-GC/MS chromatograms of wood chips degraded with the fungus *Lentinula edodes*. **a** untreated wood chips (0-wk incubation); **b** wood chips degraded for 2 wk; **c** 4 wk; **d** 8 wk; **e** 12 wk. The identities and relative abundances of the compounds represented by the numbered peaks are listed in Table 3

The pyrograms of wheat straw and wood chips during fungal treatment with *L. edodes* are shown in Figs. 1 and 2. The identities and relative abundances (mean average of three replicates) of the compounds released are provided in Table 2 (wheat straw) and Table 3 (wood chips). The pyrolysis of untreated wheat straw (Fig. 1a) and wood chips (Fig. 2a) released a similar set of compounds derived from the carbohydrate and lignin moieties, although in different proportions (Tables 2 and 3).

**Table 2:** Identities and relative abundance (mean average of three replicates) of the compounds released upon pyrolysis GC/MS of autoclaved wheat straw before and after treatment with *L. edodes* for 2, 4, 8 and 12 wk

Label	Compound	Origin^a^	Time, wk				
			0	2	4	8	12
1	(*2H*)-furan-3-one	C	3.4	4.1	4.4	4.9	5.2
2	Propanal	C	6.1	5.4	4.4	4.7	6.7
3	Furfural	C	6.0	10.1	10.4	11.2	9.7
4	2,3-dihydro-5-methylfuran-2-one	C	6.0	5.4	5.1	5.4	7.0
5	(*5H*)-furan-2-one	C	4.6	3.8	3.9	3.8	4.9
6	4-hydroxy-5,6-dihydro-(*2H*)-pyran-2-one	C	7.9	10.9	10.8	9.1	6.5
7	2-hydroxy-3-methyl-2-cyclopenten-1-one	C	3.4	3.0	3.8	5.4	5.9
8	Phenol	LH	1.1	0.9	1.0	1.1	1.0
9	Guaiacol	LG	3.4	2.5	2.4	2.2	1.6
10	3-hydroxy-2-methyl-(*4H*)-pyran-4-one	C	1.0	1.1	1.7	2.5	2.7
11	4-hydroxymethyl-1,4-butyrolactone	C	2.9	3.1	2.7	2.8	3.9
12	4-methylguaiacol	LG	1.3	1.0	0.7	0.5	0.3
13	4-ethylguaiacol	LG	0.4	0.3	0.2	0.1	0.1
14	5-hydroxymethyl-2-tetrahydrofuraldehyde-3-one	C	1.7	1.5	2.4	2.8	3.0
15	1,4-anhydroarabinofuranose	C	1.7	1.7	1.9	1.6	1.3
16	4-vinylphenol	LH/PCA	9.3	6.3	3.1	2.2	0.8
17	4-vinylguaiacol	LG/FA	8.3	5.3	2.9	2.1	0.8
18	Eugenol	LG	0.4	0.2	0.2	0.1	0.0
19	5-hydroxymethyl-2-furfuraldehyde	C	1.7	3.3	3.6	3.2	4.7
20	Syringol	LS	2.9	2.1	1.7	0.8	0.5
21	*cis*-isoeugenol	LG	0.2	0.1	0.1	0.1	0.0
22	1,4-dideoxy-d-glycerohex-1-enopyranos-3-ulose	C	0.8	1.0	1.4	2.0	1.7
23	*trans*-isoeugenol	LG	1.5	0.9	0.6	0.4	0.2
24	1,4-anhydroxylofuranose	C	2.4	2.1	3.5	2.8	2.4
25	4-methylsyringol	LS	1.4	1.2	0.7	0.2	0.1
26	Vanillin	LG	1.8	1.0	1.0	0.6	0.3
27	4-ethylsyringol	LS	0.2	0.1	0.1	0.0	0.0
28	vanillic acid methyl ester	LG	0.1	0.3	0.2	0.1	0.1
29	Acetovanillone	LG	0.4	0.5	0.5	0.5	0.3
30	4-vinylsyringol	LS	2.2	1.2	0.7	0.3	0.2
31	Guaiacylacetone	LS	0.4	0.3	0.2	0.1	0.1
32	4-allyl-syringol	LS	0.6	0.3	0.1	0.1	0.0
33	Propiovanillone	LG	0.1	0.1	0.1	0.1	0.0
34	*cis*-4-propenylsyringol	LS	0.4	0.3	0.1	0.1	0.0
35	*trans*-4-propenylsyringol	LS	2.0	1.5	0.4	0.3	0.1
36	Levoglucosane	C	10.0	15.6	21.6	25.1	27.3
37	syringaldehyde	LS	0.9	0.5	0.4	0.2	0.1
38	syringic acid methyl ester	LS	0.1	0.2	0.2	0.1	0.1
39	acetosyringone	LS	0.6	0.6	0.4	0.2	0.1
40	syringylacetone	LS	0.3	0.3	0.2	0.1	0.0
41	propiosyringone	LS	0.1	0.1	0.0	0.0	0.0
	% Lignin		40.4	27.9	18.1	12.6	7.1
	% Carbohydrates		59.6	72.1	81.9	87.4	92.9
	Lignin/Carbohydrate ratio		0.7	0.4	0.2	0.1	0.1
	H		10	7	4	3	2
	G		18	12	9	7	4
	S		12	8	5	3	1
	Syringyl/Guaiacyl ratio		0.7	0.7	0.5	0.4	0.4
	(Syringyl/Guaiacyl)_except vinyl_ ratio^b^		1.0	1.0	0.7	0.5	0.4
	Ph-C0-2/Ph-C3^c^		5.7	6.0	8.2	9.5	10.9
	% Cα-oxidized lignin		10.2	11.5	15.2	15.2	16.4
	% Cα-oxidized G-units		5.9	6.5	9.4	10.4	11.3
	% Cα-oxidized S-units		4.4	4.9	5.9	4.8	5.1

^a^C, carbohydrate-derived compounds; LH, *p*-hydroxycinnamyl lignin-derived compounds; LG, guaiacyl-lignin derived compounds; S, syringyl-lignin derived compounds; PCA, *p*-coumarates; FA: ferulates. ^b^All G- and S-derived peaks were used for the estimation of the S/G ratio, except 4-vinylguaiacol (which also arises from ferulates), and the analogous 4-vinylsyringol. ^c^Ratio of lignin-derived phenols with none, 1 and 2 carbons in the side-chain to lignin-derived phenols with 3 carbons in the side-chain

**Table 3:** Identities and relative abundance (mean average of three replicates) of the compounds released upon pyrolysis GC/MS of autoclaved wood chips before and after treatment with *L. edodes* for 2, 4, 8 and 12 wk

Label	Compound	Origin^a^	Time, wk				
			0	2	4	8	12
1	(*2H*)-furan-3-one	C	2.7	3.2	3.4	3.9	4.0
2	Propanal	C	2.5	2.1	2.2	2.2	2.2
3	Furfural	C	6.1	8.3	8.6	8.8	9.2
4	2,3-dihydro-5-methylfuran-2-one	C	4.0	3.4	3.5	4.1	3.8
5	(*5H*)-furan-2-one	C	2.6	2.7	2.5	2.7	2.4
6	4-hydroxy-5,6-dihydro-(*2H*)-pyran-2-one	C	5.5	7.8	7.3	7.1	7.4
7	2-hydroxy-3-methyl-2-cyclopenten-1-one	C	4.3	3.3	3.7	4.3	3.9
8	Phenol	LH	1.8	1.8	1.8	1.6	1.5
9	Guaiacol	LG	4.2	3.9	4.3	4.5	4.9
10	3-hydroxy-2-methyl-(*4H*)-pyran-4-one	C	1.3	1.0	1.1	1.4	1.4
11	4-hydroxymethyl-1,4-butyrolactone	C	1.1	1.0	1.0	0.7	0.8
12	4-methylguaiacol	LG	4.9	3.6	3.3	2.3	2.3
13	4-ethylguaiacol	LG	0.8	0.4	0.4	0.4	0.2
14	5-hydroxymethyl-2-tetrahydrofuraldehyde-3-one	C	1.7	1.8	2.1	2.6	2.9
15	1,4-anhydroarabinofuranose	C	0.9	0.8	0.7	0.8	0.7
16	4-vinylphenol	LH	1.0	1.0	0.7	0.7	0.6
17	4-vinylguaiacol	LG	5.8	4.9	4.4	3.2	2.5
18	Eugenol	LG	1.3	0.9	0.8	0.6	0.5
19	5-hydroxymethyl-2-furfuraldehyde	C	1.6	2.5	2.4	2.7	3.0
20	Syringol	LS	3.8	3.3	2.8	2.4	1.9
21	*cis*-isoeugenol	LG	0.9	0.6	0.5	0.3	0.2
22	1,4-dideoxy-d-glycerohex-1-enopyranos-3-ulose	C	0.4	1.3	1.3	1.3	1.6
23	*trans*-isoeugenol	LG	4.4	2.7	2.3	1.6	1.3
24	1,4-anhydroxylofuranose	C	0.8	1.2	1.1	1.3	1.4
25	4-methylsyringol	LS	3.2	2.6	1.9	1.4	1.0
26	Vanillin	LG	1.9	2.0	1.7	1.4	1.5
27	4-ethylsyringol	LS	0.6	0.4	0.3	0.2	0.1
28	vanillic acid methyl ester	LG	0.3	0.5	0.6	0.7	0.8
29	acetovanillone	LG	1.0	1.0	1.0	0.9	0.9
30	4-vinylsyringol	LS	4.4	3.5	2.4	1.8	1.3
31	guaiacylacetone	LS	0.7	0.6	0.6	0.4	0.4
32	4-allyl-syringol	LS	1.3	0.9	0.6	0.5	0.3
33	propiovanillone	LG	0.2	0.2	0.6	0.5	0.3
34	*cis*-4-propenylsyringol	LS	0.8	0.7	0.5	0.3	0.6
35	*trans*-4-propenylsyringol	LS	5.2	3.6	2.5	1.9	1.6
36	Levoglucosane	C	12.6	17.0	22.2	26.3	28.5
37	syringaldehyde	LS	1.8	1.7	1.1	0.8	0.7
38	syringic acid methyl ester	LS	0.1	0.2	0.2	0.3	0.3
39	acetosyringone	LS	0.9	0.9	0.7	0.6	0.5
40	syringylacetone	LS	0.6	0.7	0.6	0.5	0.4
41	propiosyringone	LS	0.3	0.2	0.2	0.1	0.1
% Lignin			52.1	42.7	36.6	29.9	26.9
% Carbohydrates			47.9	57.3	63.4	70.1	73.1
Lignin/Carbohydrate ratio			1.1	0.7	0.6	0.4	0.4
H			3	3	3	2	2
G			26	21	20	17	16
S			23	19	14	11	9
Syringyl/Guaiacyl ratio			0.9	0.9	0.7	0.7	0.6
Ph-C0-2/Ph-C3^b^			2.3	2.8	3.1	3.5	3.6
% Cα-oxidized lignin			12.3	15.7	16.5	17.4	19.2
% Cα-oxidized G-units			6.4	8.5	10.7	11.3	13.0
% Cα-oxidized S-units			5.9	7.2	5.8	6.1	6.2

^a^C, carbohydrate-derived compounds; LH, *p*-hydroxycinnamyl lignin-derived compounds; LG, guaiacyl-lignin derived compounds; S, syringyl-lignin derived compounds. ^b^Ratio of lignin-derived phenols with none, 1 and 2 carbons in the side-chain to lignin-derived phenols with 3 carbons in the side-chain

The relative abundances of lignin-derived phenols decreased with incubation time of wheat straw and wood chips with *L. edodes*. In the case of wheat straw degraded by *L. edodes* (Fig. 1, Table 2), the percentage of compounds released that were derived from carbohydrates upon Py-GC/MS varied from 59.6 % in the control sample (0 wk incubation time) to 92.9 % after 12 wk of incubation, while the lignin-derived phenols (H + G + S) varied from 40.4 % in the control sample to only 7.1 % after 12 wk of incubation. In the case of wood chips incubated with *L. edodes* (Fig. 2, Table 3), the percentage of carbohydrate-derived compounds released upon Py-GC/MS varied from 47.9 % in the control sample (0 wk incubation time) to 73.1 % after 12 wk of incubation, while the lignin-derived phenols varied from 52.1 % in the control sample to 26.9 % after 12 wk of incubation. The lignin/carbohydrate (L/C) ratio estimated upon Py-GC/MS decreased from 0.7 in the wheat straw control sample (0 wk incubation time) to 0.1 in the wheat straw degraded for 12 wk (Table 2), and from 1.1 in the wood chips control sample (0 wk incubation time) to 0.4 for the wood chips degraded for 12 wk (Table 3).

Among the lignin-derived phenols, the pyrograms of wheat straw show compounds derived from *p*-hydroxyphenyl (H), guaiacyl (G) and syringyl (S) lignin units, whereas lignin in wood chips contained mainly G- and S-units (Tables 2 and 3). For wheat straw, S/G ratio gradually decreased with incubation time from 0.7 in the control sample (0 wk incubation time) to a final value of 0.4 after 12 wk (Table 2). However, in wheat straw, 4-vinyl-guaiacol (compound 17) may also arise from ferulates on arabinoxylans and, therefore, the lignin S/G ratio may be underestimated [22]. Hence, a more accurate S/G ratio of the lignin in wheat straw could be obtained by ignoring 4-vinylguaiacol (compound 17) (and the analogous 4-vinylsyringol, compound 30). This S/G ratio estimated a value of 1.0 in the untreated wheat straw and shows a continuous decrease until a value of 0.4 after 12 wk of incubation (Table 2). Likewise, in the case of wood chips, a decrease of the lignin S/G ratio was also observed during fungal incubation time. The S/G ratio estimated by Py–GC/MS for the untreated wood chips sample was 0.9, and decreased from 4 wk on steadily during incubation down to a value of 0.6 in the wood chips incubated for 12 wk (Table 3). The S/G ratio in wood chips was not corrected for 4-vinylguaiacol and 4-vinylsyringol, since ferulates are abundant in grasses, but not important in wood [22].

**Fig. 3:**
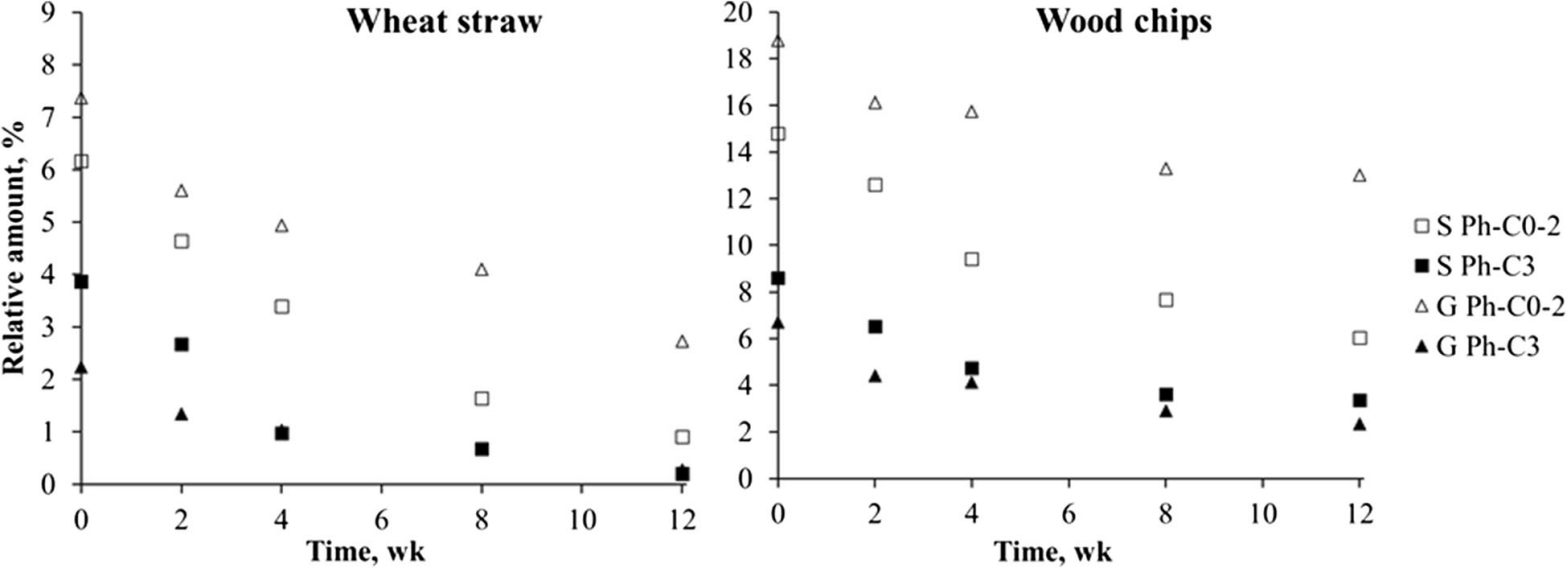
Relative amounts of Ph-C3 and Ph-C0-2 compounds originating from S- and G-units present in wheat straw and wood chips during *Lentinula edodes* treatment. □ Ph-C0-2 compounds originating from S-units ■ Ph-C3 compounds originating from S-units Δ Ph-C0-2 compounds originating from G-units ▲ Ph-C3 compounds originating from G-units

The Ph-C0-2/Ph-C3 ratio represents the ratio between lignin units with short (C0-C2) side chains (Ph-C0-2) and intact C3 side-chains (Ph-C3). The Ph-C0-2/Ph-C3 ratio in wheat straw increased from 5.7 in the untreated sample up to 10.9 in the 12-wk treated sample. In wood chips the Ph-C0-2/Ph-C3 ratio increased from 2.3 in the untreated sample up to 3.6 in the 12-wk treated sample. As seen in Fig. 3, Ph-C3 compounds originating from both S- and G-units decreased at a similar rate while Ph-C0-2 compounds originating from S-units (compounds 20, 25, 27, 30, 37, 38 and 39) are degraded faster than Ph-C0-2 compounds originating from G-units (compounds 9, 12, 13, 16, 26, 28 and 29). As a result, more Ph-C0-2 compounds originating from G-units than from S-units were found in both the fungal degraded wheat straw and wood chips, in accordance with the fact that *L. edodes* degraded more S-units than G-units.

**Fig. 4:**
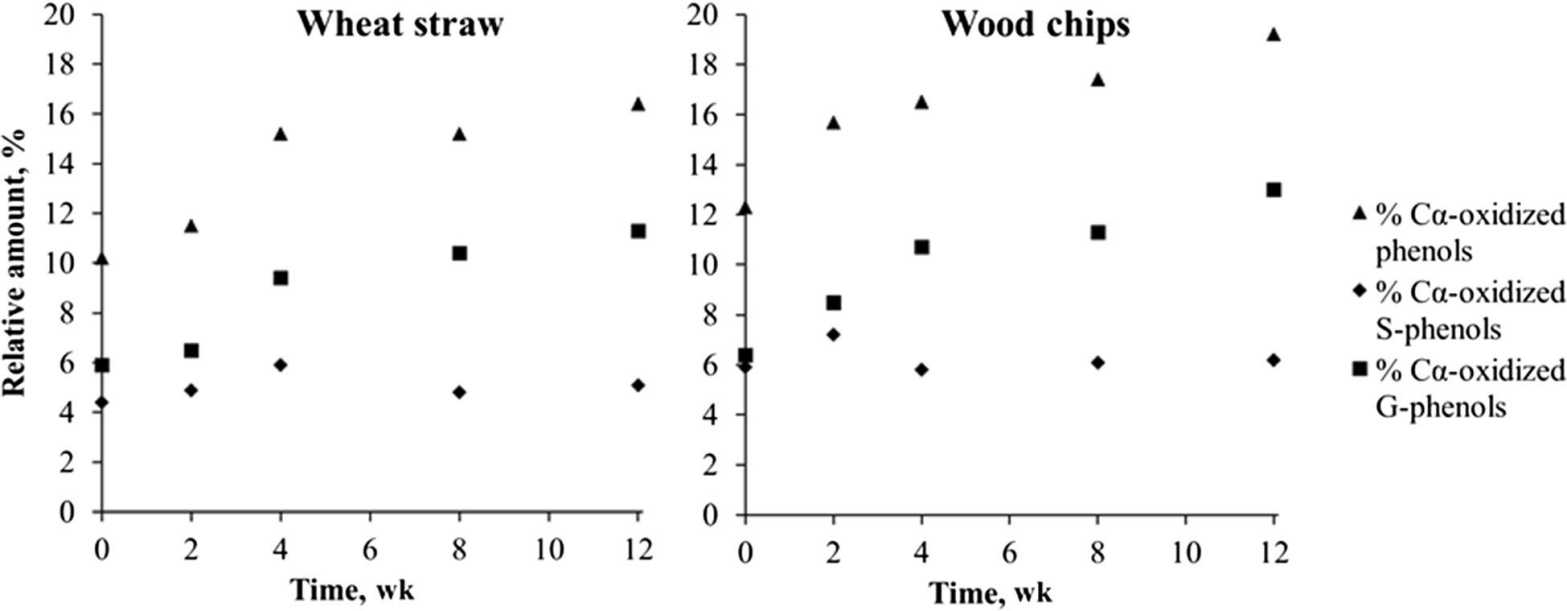
Percentage Cα-oxidized lignin units originating from S- and G-units present in wheat straw and wood chips during *Lentinula edodes* treatment. ▲total Cα-oxidized phenols ♦ Cα-oxidized products originating from S-units ■ Cα-oxidized products originating from G-units

In addition, some Cα-oxidized phenolic compounds such as aromatic aldehydes, acids and ketones were found during pyrolysis of wheat straw and wood chip samples treated with *L. edodes* (Tables 2 and 3). Among them, the relative abundance of lignin-derived compounds oxidized at the α-carbon, clearly increase after fungal treatment of wheat straw and wood chips with *L. edodes* (Tables 2 and 3). In the case of wheat straw treated with *L. edodes*, the percentage of the Cα-oxidized compounds increases continuously during fungal incubation from 10.2 % in the control sample (0 wk incubation) up to 16.4 % in the wheat straw sample after 12 wk of fungal incubation. Similarly, in the case of the wood chips treated with *L. edodes*, the percentage of Cα-oxidized compounds also increases during fungal incubation from 12.3 % in the control sample (0 wk incubation) up to 19.2 % after 12 wk of fungal degradation. Interestingly, more Cα-oxidized lignin compounds were found originating from G-lignin units (compounds 26, 28, 29 and 33) than from S-lignin units (compounds 37, 38, 39 and 41) in both wheat straw and wood chips, as reflected in Fig. 4.

### Correlations between IVGP and composition of the substrates

**Fig. 5:**
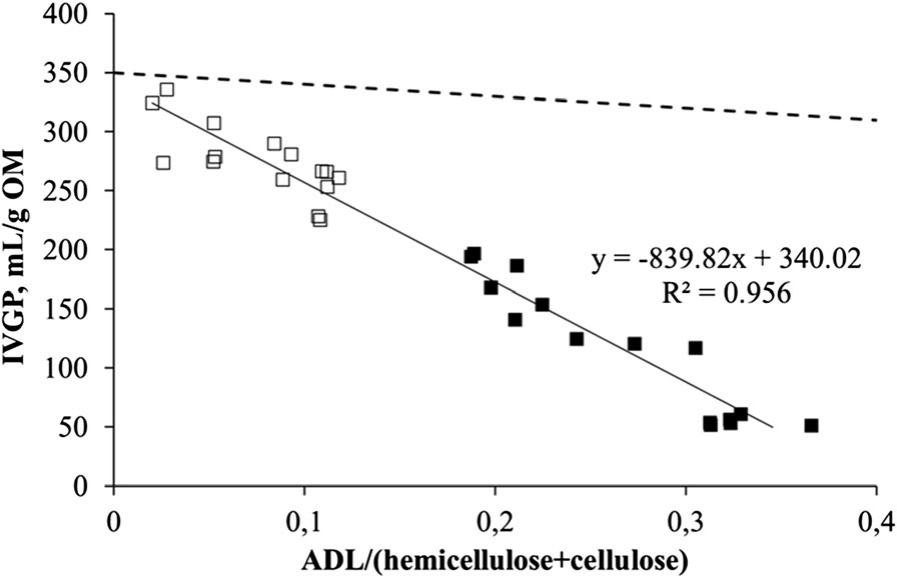
Relation between *in vitro* gas production (IVGP) and the lignin to carbohydrates ratio (lignin/hemicellulose + cellulose) as determined by the detergent fiber analysis. □ wheat straw ■ wood chips, dashed line: potential maximum IVGP

IVGP showed a linear relationship to changes in cell wall composition (ADL to carbohydrate (hemicellulose + cellulose) ratio) as determined by the detergent fiber analysis (Fig. 5). This relation was similar for both wheat straw and wood chips.

**Fig. 6:**
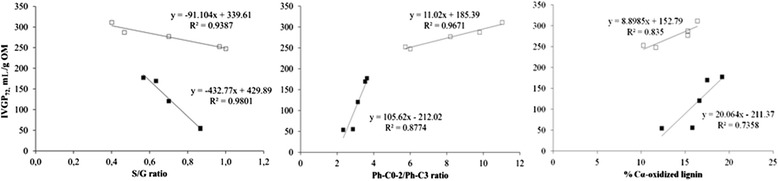
Correlations between *in vitro* gas production (IVGP) and lignin/carbohydrate ratio, S/G ratio, Ph-C0-2/Ph-C3 ratio and %Cα-oxidized lignin. □ wheat straw ■ wood chips

The increase in IVGP of fungal treated wheat straw was negatively correlated with the L/C ratio (*r* = −0.69, *P* < 0.01) determined by Py-GC/MS (Fig. 6). S/G ratio was also negatively correlated with IVGP (*r* = −0.72, *P* < 0.01), while a positive correlation was found between IVGP and the percentage of Cα-oxidized lignin compounds (*r* = 0.77, *P* < 0.01) and the Ph-C0-2/Ph-C3 ratio (*r* = 0.51, *P* = 0.05) determined by Py-GC/MS (Fig. 6). Similar to wheat straw, IVGP of fungal treated wood chips was also negatively correlated to the L/C ratio (*r* = −0.88, *P* < 0.01) and S/G ratio (*r* = −0.75, *P* < 0.01) (Fig. 6), while a positive correlation between IVGP of wood chips and Ph-C0-2/Ph-C3 ratio (*r* = 0.77, *P* < 0.01) and %Cα-oxidized lignin (*r* = 0.62, *P* = 0.01) was found (Fig. 6).

## Discussion

Fungal incubations using *L. edodes* were used as a pre-treatment for wheat straw and wood chips for their subsequent use as ruminant feed. The fungal incubations were done at 24 °C to make it comparable to earlier studies. However, it should be noted that the optimal temperature for *L. edodes* may be higher, meaning that a faster colonization and delignification could be achieved. To make sure only *L. edodes* was present in the culture, the substrates were sterilized before inoculation. In this study, a 2 h cycle was used to eliminate the spores. However, autoclaving for 2 h may compare to a severe thermal treatment, which may result in changes in the cell wall. For this reason, a less severe sterilization step before fungal inoculation should be used in future studies. Also, sterilization causes a loss in moisture. The moisture content in this study was controlled by incubation in a room with 70 % relative humidity. Because of the small size of the containers (1.2 L), which are covered with a lid, it is expected that moisture loss upon incubation is minimal. Regression analysis showed that changes in ADL content influence changes in IVGP most. This is in line with a previous paper [4] that found that ADL content and IVGP were negatively correlated. ADL represents the non-degradable fraction that is defined as lignin [11]. However, it does not include acid soluble lignin, and due to a filtration step, it might not include smaller fragments of lignin (such as degradation products) [12, 13]. The detergent fiber analysis method used in the current study used filter bags with pore size of 25 μm, meaning that smaller, unbound compounds will be lost during analysis. Also, fungal biomass might be analyzed as ADL, since Jurak et al. [23] also observed a Klason lignin fraction in button mushrooms. To determine the effect of lignin composition, without contamination of the lignin fraction with fungal biomass, lignin was studied in more detail using Py-GC/MS. A preferential lignin degradation occurred in both substratesproducing a residue enriched in cellulose, which was more pronounced in wheat straw. These results confirm the preferential lignin degradation found by *L. edodes* in beech and wheat straw [4, 17]. The higher lignin degradation found in wheat straw compared to wood chips is likely due to the higher lignin content in wood (L/C ratio of 0.3) compared to wheat straw (L/C ratio of 0.1) and possibly to physical differences in the two types of substrate (such as density of tissues and the surface to content ratio). It must be noted, however, that the L/C estimated by Py-GC/MS ratios do not reflect the real content of each moiety since pyrolysis is known to be more sensitive for lignin as the cellulose is significantly underestimated due to intense charring and extensive degradation to non-chromatographed products [20]. However, the L/C ratios observed upon pyrolysis can still be used for comparison of the relative amounts of individual moieties of lignocellulose in the analyzed samples and visualize the direction in which compound concentrations changes. For future studies it is advised to include a carbohydrate analysis to study the fate of these compounds more in detail. The lignin degradation is confirmed by the increasing occurrence of lignin compounds with shorter side chains (Ph-C0-2) and the occurrence of Cα-oxidized lignin compounds. Interestingly, Ph-C0-2 compounds were also detected in the untreated, autoclaved control. These compounds are generated during the pyrolysis of condensed lignin structures. Since G-units form more condensed structures, more Ph-C0-2 compounds originating from G-lignin were found (Fig. 3).

The Py-GC/MS data showed a change in lignin composition by *L. edodes*. Two different phases in lignin composition changes by *L. edodes* can be defined. The first phase is characterized by radical attack of lignin, since lignin degrading enzymes cannot enter the intact cell walls during the initial phase of fungal delignification [24]. These radicals are likely less specific resulting in a simultaneous degradation of S- and G-units during the first 2 to 4 wk of *L. edodes* treatment. On the other hand, lignin side-chain oxidation does occur at Cα under the influence of both the radicals and direct enzymatic degradation [24], resulting in an increased percentage of Cα-oxidized lignin units released upon Py-GC/MS within the first 2 wk of *L. edodes* treatment as was also shown for other fungal treated lignocellulosic samples [16, 18]. During the second phase, the final stages of the fungal treatment, lignin degradation shifts from radical degradation toward enzymatic degradation [17]. With enzymatic degradation preferential S- over G-unit degradation starts after 4 wk of *L. edodes* treatment. In wood chips, this preferential S-unit degradation is accompanied by a significant increase in IVGP. In the calculation of the S/G ratio for wheat straw, 4-vinylguaiacol (compound 17) and 4-vinylsyringol (compound 30) were excluded. The presence of these compounds is mostly due to the presence of *p*-coumarates and ferulates, which decarboxylate efficiently under pyrolytic conditions producing these vinyl compounds, as previously shown [25]. The decrease of 4-vinylguaiacol (compound 17) in wheat straw suggests a degradation of ferulates or the arabinoxylans to which ferulates are bound. This result is in accordance with the hemicellulose degradation found by the detergent fiber method. The increase in the relative amounts of partly degraded G-units at a later stage of fungal degradation might indicate that S-units are degraded further and are not detected anymore, which is in accordance with the preferential S-unit degradation by *L. edodes*.

The higher biodegradability of the S-lignin compared to the G-lignin has already been shown in several other studies showing the decrease of the S/G ratio during degradation of other lignocellulosic substrates by different white-rot fungi [9, 16–18, 26, 27]. S-lignin units are more prone to enzymatic fungal degradation because they have a higher predominance of β-*O*-4 ether linkages compared to G-units that are more recalcitrant to fungal attack due to the formation of condensed linkages [18]. However, it has recently been indicated that S-rich transgenic poplar woods exhibited improved resistance to fungal degradation [28], which suggests that besides composition of lignin, other features such as the 3D-structure of cell walls is important in degradation. To confirm this, future studies should include additional 2D-NMR analysis to obtain information about linkanges within the cell wall.

Here, the S/G ratio is correlated to IVGP (Fig. 6), suggesting that lignin composition has an influence on rumen degradability. Changes in S/G ratio of wood chips have a larger influence on rumen degradability than changes in S/G ratio of wheat straw. A similar trend for Ph-C0-2/Ph-C3 ratio was found in wood chips and wheat straw (Fig. 6). In the current study, with the degradation of lignin, also its structure, composition and content are changing. The relatively large effect of changes in S/G ratio and Ph-C0-2/Ph-C3 ratio are related to the high lignin content of wood chips. Small changes in lignin will have a relatively large effect on accessibility of rumen microbes. However, there is a high linear correlation between the L/Cratio and IVGP (Fig. 5), where both substrates with different lignin composition show an identical trend. This indicates that lignin content, which was different between wheat straw and wood chips, is more important than lignin composition, which does not have any effect on IVGP. The latter is in line with previous works that reported no effect of lignin composition on the degradation of polysaccharides in maize cell walls by enzymes of *Trichoderma reesei* and *Aspergillus niger* or rumen microbes [29]. It is important to note that lignin degradation started during the first 2 to 4 wk, while both S/G ratio and IVGP did not change. This suggests that lignin structure, i.e., the binding to carbohydrates, rather than lignin composition is important for rumen degradability. Access of rumen microbes to the fermentable carbohydrates seems to be physically blocked by the presence of bound lignin in the cell wall matrix. This is demonstrated by the theoretical potential maximum IVGP if all ADL would be removed (dashed line in Fig. 5). The maximum IVGP will be approximately 350 ml/g OM, since removal of total ADL will result in pure carbohydrates. The maximum IVGP decreases slightly, since ADL is diluting the carbohydrates, i.e., at a higher ADL/(hemicelluloses + cellulose) ratio a lower amount of carbohydrates are present (Fig. 6). This suggests that both the content of lignin and composition of lignin (S/G ratio) are not influencing IVGP during the first 2 to 4 wk. Probably the linkages between lignin and carbohydrates [30] and the 3D-structure of lignin block the rumen microbes. If this is true, removal of the linkages between lignin and carbohydrates would result in a theoretical IVGP of approximately 350 ml/g OM (dashed line in Fig. 5). This value would decrease with an increasing lignin/carbohydrate ratio, because of the diluting effect of lignin. This theoretical IVGP is only true under the assumption that lignin is not toxic for rumen microbes.

Diverse and complex products are generated during degradation of lignin by *L. edodes*. Phenolic compounds originating from lignin such as cinnamic acid and vanillin are described to inhibit cellulose degradation by rumen microbes [31], but it should be noted that in scientific literature these compounds were added in pure form. In contrast, when these compounds are present in the matrix of fungal treated biomass, they do not inhibit IVGP as observed in the current study. Also, other studies describing fungal treatment of lignocellulose found increased *in vitro* rumen degradation, suggesting that degradation products from fungal treatment do not inhibit rumen microbes [4–6].

## Conclusion

In this study, changes in lignin composition were a direct result of lignin degradation since it was mainly related to the mechanisms of fungal degradation and less to substrate properties. The main results were selective S-unit over G-unit degradation and side chain degradation of lignin. The changes in lignin had a similar effect on IVGP of wheat straw and wood chips. It is concluded that lignin content and the 3D-structure of cell walls have a larger influence on *in vitro* rumen degradability than changes in lignin composition.

## Abbreviations

ADF: Acid detergent fiber
ADL: Acid detergent lignin
DM: Dry matter
GLM: Generalized linear model
GRAS: Generally regarded as safe
G-unit: Guaiacyl unit
H-unit: *p*-hydroxyphenyl unit
IVGP: *In vitro* gas production
NDF: Neutral detergent fiber
OM: Organic matter
S-unit: Syringyl unit

## References

[1] Ahmad M, Taylor CR, Pink D, Burton K, Eastwood D, Bending GD (2010). Development of novel assays for lignin degradation: comparative analysis of bacterial and fungal lignin degraders. Mol Biosyst.

[2] Sarnklong C, Cone JW, Pellikaan W, Hendriks WH (2010). Utilization of rice straw and different treatments to improve its feed value for ruminants: a review. Asian-Aust J Anim Sci.

[3] van Kuijk SJA, Sonnenberg ASM, Baars JJP, Hendriks WH, Cone JW (2015). Fungal treated lignocellulosic biomass as ruminant feed ingredient: a review. Biotechnol Adv.

[4] Tuyen VD, Cone JW, Baars JJP, Sonnenberg ASM, Hendriks WH (2012). Fungal strain and incubation period affect chemical composition and nutrient availability of wheat straw for rumen fermentation. Bioresour Technol.

[5] Tuyen DV, Phuong HN, Cone JW, Baars JJP, Sonnenberg ASM, Hendriks WH (2013). Effect of fungal treatments of fibrous agricultural by-products on chemical composition and *in vitro* rumen fermentation and methane production. Bioresour Technol.

[6] Permana IG, ter Meulen U, Flachowsky G, Zadrazil F. Cultivation of *Pleurotus ostreatus* and *Lentinus edodes* on lignocellulose substrates for fruiting bodies and animal feed production. Deutscher Tropentag 2000.

[7] Gaitán-Hernández R, Esqueda M, Gutiérrez A, Sánchez A, Beltrán-García M, Mata G (2006). Bioconversion of agrowastes by *Lentinula edodes*: the high potential of viticulture residues. Appl Microbiol Biotechnol.

[8] Lin Y, Ge X, Liu Z, Li Y (2015). Integration of Shiitake cultivation and solid-state anaerobic digestion for utilization of woody biomass. Bioresour Technol.

[9] Vane CH, Drage TC, Snape CE (2003). Biodegradation of oak (*Quercus alba*) wood during growth of the shiitake mushroom (*Lentinula edodes*): a molecular approach. J Agric Food Chem.

[10] Zhao X, Gong J, Zhou S, OuYang K, Song X, Fu C, Xu L, Qu M (2015). Effect of fungal treatments of rape straw on chemical composition and in vitro rumen fermentation characteristics. Bioresour.

[11] Van Soest PJ, Robertson JB, Lewis BA (1991). Methods for dietary fiber, neutral detergent fiber, and nonstarch polysaccharides in relation to animal nutrition. J Dairy Sci.

[12] Jung H-JG (1997). Analysis of forage fiber and cell walls in ruminant nutrition. J Nutr.

[13] Godin B, Agneessens R, Gerin P, Delcarte J (2014). Structural carbohydrates in plant biomass: correlations between the detergent fiber and dietary fiber methods. J Agric Food Chem.

[14] Ralph J, Lundquist K, Brunow G, Lu F, Kim H, Schatz PF (2004). Lignins: natural polymers from oxidative coupling of 4-hydroxyphenylpropoids. Phytochem Rev.

[15] Vanholme R, Demedts B, Morreel K, Ralph J, Boerjan W (2010). Lignin biosynthesis and structure. Plant Physiol.

[16] del Río JC, Gutiérrez A, Martínez MJ, Martínez AT (2001). Py-GC/MS study of *Eucalyptus globulus* wood treated with different fungi. J Anal Appl Pyrolysis.

[17] Faix O, Bremer J, Schmidt O, Stevanovic JT (1991). Monitoring of chemical changes in white-rot degraded beech wood by pyrolysis-gas chromatography and Fourier-transform infrared spectroscopy. J Anal Appl Pyrolysis.

[18] del Río JC, Speranza M, Gutiérrez A, Martínez MJ, Martínez AT (2002). Lignin attack during eucalypt wood decay by selected basidiomycetes: a Py-GC/MS study. J Anal Appl Pyrolysis.

[19] Faix O, Meier D, Fortmann I (1990). Thermal degradation products of wood. Holz Roh Werkst.

[20] Ralph J, Hatfield RD (1991). Pyrolysis-GC-MS characterization of forage materials. J Agric Food Chem.

[21] Cone JW, van Gelder AH, Visscher GJW, Oudshoorn L (1996). Influence of rumen fluid and substrate concentration on fermentation kinetics measured with a fully automated time related gas production apparatus. Anim Feed Sci Technol.

[22] del Río JC, Prinsen P, Rencoret J, Nieto L, Jiménez-Barbero J, Ralph J (2012). Structural characterization of the lignin in the cortex and pith of elephant grass (*Pennisetum purpureum*) stems. J Agric Food Chem.

[23] Jurak E, Punt AM, Arts W, Kabel MA, Gruppen H (2015). Fate of carbohydrates and lignin during composting and mycelium growth of *Agaricus bisporus* on wheat straw based compost. PLoS ONE.

[24] Martínez AT, Speranza M, Ruiz-Dueñas FJ, Ferreira P, Camarero S, Guillén F (2005). Biodegradation of lignocellulosics: microbial, chemical, and enzymatic aspects of the fungal attack of lignin. Int Microbiol.

[25] del Río JC, Rencoret J, Prinsen P, Martínez AT, Ralph J, Gutiérrez A (2012). Structural characterization of wheat straw lignin as revealed by analytical pyrolysis, 2D-NMR, and reductive cleavage methods. J Agric Food Chem.

[26] Valmaseda M, Martínez MJ, Martínez AT (1991). Kinetics of wheat straw solid-state fermentation with *Trametes versicolor* and *Pleurotus ostreatus* - lignin and polysaccharide alteration and production of related enzymatic activities. Appl Microbiol Biotechnol.

[27] Choi JW, Choi DH, Ahn SH, Lee SS, Kim MK, Meier D (2006). Characterization of trembling aspen wood (*Populus tremuloides* L.) degraded with the white rot fungus *Ceriporiopsis subvermispora* and MWLs isolated thereof. Holz Roh Werkst.

[28] Skyba O, Douglas CJ, Mansfield SD (2013). Syringyl-rich lignin renders poplars more resistant to degradation by wood decay fungi. Appl Environ Microbiol.

[29] Grabber JH, Ralph J, Hatfield RD, Quideau S (1997). *p*-Hydroxyphenyl, guaiacyl, and syringyl lignins have similar inhibitory effects on wall degradability. J Agric Food Chem.

[30] Grabber JH, Mertens DR, Kim H, Funk C, Lu F, Ralph J (2009). Cell wall fermentation kinetics are impacted more by lignin content and ferulate cross-linking than by lignin composition. J Sci Food Agric.

[31] Varel VH, Jung H-JG (1986). Influence of forage phenolics on ruminal fibrolytic bacteria in in vitro fiber degradation. Appl Environ Microb.

